# Better Together? Analyzing Experiences from Male and Female Students and Teachers from Single-Sex and Coeducational Physical Education Classes

**DOI:** 10.3390/bs12090306

**Published:** 2022-08-25

**Authors:** Anika Frühauf, Franziska Hundhausen, Martin Kopp

**Affiliations:** Department of Sport Science, University of Innsbruck, 6020 Innsbruck, Austria

**Keywords:** school, exercise, stereotypes, gender, qualitative interviews

## Abstract

Purpose: Since personal, environmental and behavioral factors influence students’ and teachers’ actions and experiences, the present study aimed to assess gender-related experiences of students and teachers from single-sex and coeducational physical education (PE) settings. Method: In total, 64 students (mean age: 13.8 ± 0.5 years) and 12 PE teachers from single-sex and coeducational PE settings from higher education schools (6th to 13th grade) in Germany and Austria were interviewed. Interviews were analyzed using a thematic content approach. Results: Students from coeducational PE settings described more gender-unrelated behavior and a higher variety of activities including various non-gender conforming activities. Male students from single-sex PE settings reported performing only gender conform and some gender-neutral activities. Teachers from coeducational settings stated that they purposefully formed mixed-gender groups to foster social processes. A lack of time and lack of knowledge were named by PE teachers as reasons for not addressing gender issues in PE by teachers. Conclusion: Performed activities and teachers’ behavior differed between PE settings, eventually influencing reported differences in terms of behavior and gender perception by students. Further studies should evaluate the influence of specific physical activity interventions on gender perceptions and students’ behavior in order to give practical recommendations for PE classes.

## 1. Introduction

Regular physical activity reduces mortality risk and positively affects cognitive ability and mental health [1]. During adolescence, it can reduce the risk of depression in adulthood [2]. Nevertheless, 81% of those aged 13–15 years, do not reach the recommended level of 60 min of moderate-to-vigorous physical activity (MVPA) per day [3].

Physical education (PE) in schools can increase physical activity levels in adolescents [4]. However, motivational factors of PE seem to be dependent on various factors such as teaching style, type of activity and PE setting (e.g., [5,6]). PE settings, such as single-sex or coeducational classes seem to affect physical activity and well-being, especially in girls [5]. The PE setting usually depends on the regulation of the specific country. In the United States, mixed-sex classes, which are also known as coeducational classes, have been promoted by law for 50 years and include all subjects (Title IX of the Education Amendments Act of 1972; 20 U.S.C. §1681–§1688). Whereas in Austria, primary schools are usually coeducational; the law foresees physical education as separated by sex beginning in fifth grade (‘Schulorganisationsgesetz [school organization act] §8b. (1) BGBI. Nr. 242/1962’).

Single-sex classes are at risk of increasing gender differences by reproducing gender stereotypes [7,8]. Children in early and late adolescence are aware of gender biases. Girls were more aware of gender bias than boys and also were more likely to give an example of being a target of gender bias [7]. Brown and colleagues showed that girls mostly referred to sports as an example of gender bias (e.g., ‘boys being more athletic’ and ‘girls won’t be picked by teams’), whereas boys more often referred to teachers’ differing perceptions between girls and boys (e.g., ’teachers think that girls are smarter and more mature’) [7]. To shed more light on the potential differences related to gender biases, the present study tried to compare the experiences of students and teachers from single-sex and coeducational physical education (PE) settings.

### 1.1. Theoretical Framework

As a theoretical framework, this study is guided by the social cognitive theory of gender development and differentiation [9]. Gender development is influenced by an interplay between personal (biological, cognitive and affective), environmental (of the social and institutional environment) and behavioral factors. The personal contribution refers to gender-linked perceptions, behavioral and judgmental norms but also the self-regulatory influences one person has. The behavioral factor refers to activity patterns linked to gender and the environmental factor refers to a variety of social influences in daily life (e.g., family, institution, media). The interaction and weighting of factors are dependent on social systems, activities, situations and opportunities and can change throughout the life course. Observational learning is seen as the process most influencing gender development [9].

### 1.2. The Social Construction of Gender in Sport

Sport is discussed to enhance hegemonic masculinity [10], but in the last decades, women’s participation in sports has markedly risen. For instance, the proportion of female athletes in the Olympic Games has largely increased from 14.6% in 1972 to 45% in 2016 [11,12]. However, men are still more active than women in 94% of the countries (where data are available) including high-income countries [12]. The domain of sport has mostly kept its conservative role in gender relations [13] which is shown through sex segregation throughout sports participation in differentiated spaces, rules and gendered sports activities. For example, male gender-conform sports are named as soccer and fighting, whereas dancing and gymnastics are perceived as feminine and swimming and tennis as neutral [14]. Those findings appear cross-cultural in the USA, Sweden and France and across the age starting with kindergarten children [14]. Reasons for those perceptions were named by children (8–10 years) in the study of Schmalz and Kerstetter as observational issues in the environment and media (“..on TV all I see is guys on their team.” ([15], p. 548)) and stereotypes (e.g., ‘..because boys like to get dirty’ ([15], p. 550)). This suggests that stereotypes of activities and gender roles associated with these sports are internalized early during childhood and shared across western countries [14].

### 1.3. Literature Review on PE Setting and Gender

Single-sex physical education settings were reported to be providing physiological and psychological benefits, especially for girls. Studies subsequently showed that girls in single-sex settings had higher moderate-to-vigorous physical activity levels [5,16], higher enjoyment and perceived competence in PE [5,17], as well as a higher self-concept and better grades in PE [17] compared to girls in coeducational settings. However, contradicting results of physical activity and motivation for PE in girls in coeducational settings were also reported [18,19].

Environmental factors in the physical education context are named as PE setting, lesson content and teacher behavior [20]. Teacher behavior can influence and reconstruct social relations on gender and ethnicity in PE [21]. Reflecting on their class management might enable teachers to rethink their practice and personal assumptions [21].

Lesson context, investigated as gameplay and skill drills, was a determinant of activity levels in PE [18]. It was shown that boys showed similar activity levels in both single-sex and coeducational PE contexts, but girls showed higher activity levels in coeducational PE. Girls-only classes had significantly more skill drills and less gameplay resulting in reduced activity levels [18]. Korfball, which was developed as a mixed-sex game, showed that activity level was significantly higher in coeducational than single-sex classes and that unlike previous research suggested, girls showed higher activity levels compared to boys in coeducational settings [20].

However, not only research on physical activity levels provided contradictory results to the aforementioned advantages of single-sex classes for girls, but also motivation for PE was shown to be affected by class sex composition. In a Suisse study, using a within-subject intervention design with five technical physical activity tasks, motivation for task development was higher in a coeducational setting compared to a single-sex setting irrespective of the sex of the students [19].

Although the curriculum is based on inclusivity, gender stereotypes about traditional gender-conform sports, gender roles and male and female behavior were still present in PE classes and PE teachers [22,23]. In a recent review on empirical German language research in physical education, gender was identified as a low-researched topic with only 4 out of 160 included articles assessing this topic [24].

Since there seem to be multiple factors that influence students’ actions and experiences in PE, the present study aimed to qualitatively assess the experiences and attitudes of students and teachers from single-sex and coeducational PE settings to get a better understanding of the gendered experiences and implementation of gender aspects in different PE settings.

## 2. Materials and Methods

The current research was guided by a pragmatic research philosophy [25]. Pragmatism acknowledges that multiple realities exist and focuses on the research problem and the consequences [25,26]. Pragmatic research is used in sport and exercise psychology and allows the use of pluralistic approaches to gain a deeper understanding of the phenomena of interest [26,27]. Following the research aim and in line with the philosophical approach, a qualitative approach using semi-structured interviews was applied.

### 2.1. Participants

In total, 64 students from public higher education schools in Germany and Austria were interviewed. Data saturation is expected after 20–30 interviews [28], resulting in 33 students from single-sex PE settings and 31 students from coeducational PE settings. Further, 12 teachers, 6 from coeducational and 6 from single-sex education were interviewed. Participants were selected using a combination of purposive sampling strategies, namely criterion-based [29] for PE teachers and snowball recruitment for students [30]. Students of interviewed PE teachers were asked to participate in the study. The interviews took place during school hours and had a duration of 10–20 min. Five to a maximum of ten students were interviewed from each participating class. The primary inclusion criterion was that PE teachers were either from a single-sex or coeducational setting and taught physical education for at least one year prior to the interview. Since physical education is a compulsory subject in both Germany and Austria, the primary inclusion criterion for students was to attend sixth grade or higher. It was ensured to include an even distribution of gender and education settings for both teachers and students.

### 2.2. Procedure

A semi-structured interview was carried out with each participant. An interview guide was used to ensure that each participant was asked the same questions but at the same time allow them to talk freely about their experiences. The interview guide for students included questions on performed activities and how students coped with performing non-gender conform activities (e.g., which activities have you performed during PE classes? How do you deal with performing non-gender conform activities?). Further questions were asked regarding gender stereotypes and their handling of it in PE (what is typical boys’ and girls’ behavior in PE? Do you address gender stereotypes in PE?). Teachers were asked which gender roles they notice during PE and how students react when having to perform non-gender conform activities. Further, teachers were asked about their gender-sensible teaching methods: what they know about gender-sensible teaching methods, how they address gender bias in their PE classes and further if they can observe socialization processes in their PE classes. Interviews were carried out by one interviewer (FH). All interviews were conducted in German and carried out one-to-one, with three exceptions, where two students were interviewed parallel since they preferred not talking alone with the interviewer. Participation was voluntary with five to a maximum of ten students participating from each class. The study was approved by the head of the schools and the participating teachers allowed the interviewed students to leave the classroom for the duration of the interviews. 

### 2.3. Analysis

Before analyzing the data, all interviews were transcribed verbatim by the interviewer. Transcription was carried out immediately after the interview. Any non-verbal communication was noted such as laughs or long pauses. The data were then analyzed independently by the first author, who was not the interviewer, in several distinct stages using MAXQDA Software (“MAXQDA,” 1995–2022). Firstly, the interviews of the students were analyzed where the first author read the transcripts several times to immerse in the data as a first step. Secondly, an inductive thematic content analysis [31] was carried out where raw data were given codes (e.g., “aggressive”). This procedure was repeated for all 64 interviews of the students. In the next step of the analysis, all interviews were cross-checked, ensuring that coding was consistent and accurately represented the data. Following this, similar codes were grouped into subthemes (e.g., ‘boys behavior’) and higher-order themes (e.g., ‘Stereotypes in behavioral attributes’).

The final step was to confirm the codes and themes with the co-authors, whereas the last author acted as a critical friend [32]. Although the critical friend did not analyze the full sample, in terms of disagreement or uncertainty by the primary analyst, codes and themes were discussed until full agreement was reached. The same procedure was applied to the interviews of the teachers. In addition to this, raw quotes have been presented in English, with the hope that the data will speak for itself and the voices of the participants might be heard. The participants were indicated by numbers (1–63), gender (f, female; m, male) and PE setting (si, single-sex PE setting; co, coeducational PE setting). The reference 11f_co, for example, indicates a female student from a coeducational setting. Teachers are further identified in brackets (e.g., Teacher 6m_co).

## 3. Results

### 3.1. Sample Characteristics

In total, 64 students from three different public higher education schools were interviewed. All schools were located in an urban area. The students were part of grades 6–13 and aged between 11–18 years (Table 1). Further, 12 teachers from the same schools were interviewed; 6 teachers (37.9 ± 9.9 years) teaching single-sex (4 female, 2 male) and 6 teachers (2 female, 4 male) teaching coeducational PE.

### 3.2. Main Results

Three higher-order themes emerged from the analysis of students’ and teachers’ interviews: stereotypes in behavioral attributes, gendered sports activities and reported difficulties. A fourth higher-order theme, gender-sensible teaching, only refers to teachers’ interviews.

Gender stereotypes were reflected in the students’ descriptions of gendered behavior and sports activities. Differences between single-sex and coeducational PE settings were mainly seen in the description of stereotypical behavior. Half of the coeducational students (50%) and only two students from single-sex educational settings (1%) described a neutral gender behavior; they did not think that specific attributes were only valid for boys or girls. Practiced activities differed between single-sex and coeducational PE settings. Boys from single-sex PE only performed gender conform or neutral-classified sports during PE, whereas students from coeducational PE settings performed a high variety of gender conform and non-gender conform sports. Furthermore, 30% of coeducational and only 1% of single-sex PE settings perceived sports as gender-neutral and did not link certain sports to a specific gender.

#### 3.2.1. Stereotypes in Behavioral Attributes

A neutral gender behavior was described by 31 students from coeducational classes and 2 students from single-sex classes. As the following quotes illustrate, students from coeducational classes did not think that a specific behavior could be attributed to a gender: “Well, there is not really something that only boys do or only girls do, it’s very mixed.” (8m_co). One girl explained that behavior in PE depended on the leisure time physical activity and not on gender: “But you can see that regardless of gender, those who play team sports in their leisure time like basketball, soccer, handball, they are all always somehow more involved [in PE].” (30f_coed).

The most common attributes of stereotypical behavior in girls were named as girls being reserved and/or cautious, afraid to get hit by a ball and not motivated for PE (Table 2) as the following quote illustrates: “So what I’ve noticed is that in ball sports, so I personally know many [girls], who are afraid of the ball, especially when there’s a 1.80 meter guy who has a lot of strength and you’ll get hit on your fingers, that hurts. Many [girls] are very restrained and that was already the case in elementary school, so it was always the case when it was about playing with balls, the girls were gone.” (31f_co).

Although those descriptions were named by boys and girls, more girls than boys named those attributes. A difference between PE settings was seen in the description of ‘afraid to get hit by a ball’ which was named by more students from coeducational classes and in ‘not motivated for PE’ which was named by more students from single-sex classes.

“But I’ve noticed it with our girls, they don’t have such a competitive spirit, for them winning isn’t the best thing. They’re also quick to snap when you yell and then we’ve already had a few conflicts, I say now, because there are rather few who really want to win. Some are just standing around and that’s unfortunately really typical of the girls [in our PE class], that there is no competitive spirit or ambition and they’re so sensitive, that’s actually quite strongly represented in our PE group, unfortunately yes.”

(4f_si)

Boys were described as being posers, loud, egoistic and aggressive. The attribute of boys being egoistic was named by more girls than boys and by coeducational and single-sex PE settings equally. Being egoistic usually referred to the experience of team sports when boys did not want to pass the ball. One girl described behavioral differences between boys and girls which did not necessarily lead to better performance:

“When playing football, for example, I often think that boys play much rougher and more physical I would say, and well, but I don’t think that boys are much better than girls, although some people always say that, but I think that it is relatively equal. But boys are often louder than girls.”

(11f_co)

All other descriptions regarding boys’ behavior in PE were made by more boys than girls and more students from single-sex settings than coeducational settings. The biggest difference was seen in the attribute aggressive which was only named by students from single-sex PE settings as described by this student “Sometimes, or I say it like this—in soccer, it’s that if somebody makes a fault, then a guy gets aggressive and you’ll get yelled at.” (57m_si).

##### Teachers’ Views

Teachers partially confirmed descriptions of students’ behavior. They described girls as being more reserved and cautious, but that behavior in PE classes also depended on personality and athleticism. Boys’ behavior was described as more dominant and competitive (also in comparison to girls). Some noticed more aggressive behavior in boys as this teacher explained:

“Boys are more aggressive in their behavior. So more often aggressive when something doesn’t work out, so the frustration tolerance is perhaps not as high as with the girls, they are perhaps not quite as dogged, but boys are sometimes also more willing to perform. Well, these are stereotypes, of course there are always girls who are totally willing to perform and ambitious, but these are the stereotypes that I have noticed so far.”

(teacher 8f_co)

Boys were further described as more motivated for PE which one teacher attributed to the time they need to get ready for PE. However, not all teachers reported visible gender roles in their PE classes. One coeducational teacher reported that gender roles were depending on the school and that he did not see any gender differences worth mentioning at the moment:

“No difference, so nothing worth mentioning. At the old school, it was really the case that girls stopped participating as soon as they started sweating, and that’s not the case here at all, they don’t care. They always have fun anyway.”

(Teacher 6m_co)

All interviewed single-sex PE teachers only taught same-sex classes (e.g., female teachers taught girls-only classes) and only had rare experiences of teaching opposite-sex classes as a substitute. Thus, experiences regarding gender comparisons were not made by single-sex teachers.

#### 3.2.2. Gendered Sport Activities

Practiced sports differed between students from single-sex and coeducational PE settings. Students from coeducational PE settings reported having practiced a wide range of sports such as acrobatics, floorball, frisbee, soccer (variations), wrestling, rope jumping, parkour, hockey, badminton, American football, handball, rugby and dance forms (e.g., jumpstyle). None of the students from coeducational PE settings reported having disliked non-gender conform activities. Some boys from coeducational PE settings were reported to be reserved at the beginning when a non-gender conform sport was introduced such as the following two quotes of coeducational male students explain: “It didn’t really matter whether it was more for girls or boys -in retrospect, it was fun anyway.” (17m_co).

“So that [performing a non-gender conform sport] was actually kind of unpleasant at the beginning. […] We used to have aerobics and many exercises which are not what you would call super masculine and well you have to somehow cross an inhibitory threshold but then it actually works.”

(27m_co)

Female students usually liked to practice non-gender conform sports as this student explained: “I think it‘s good [trying non-gender conform sports], because then nobody can say that it‘s only a boys sport. I don‘t think there are any boys’ or girls’ sports although they might be represented more by one gender.” (11f_co).

Students from single-sex PE settings mostly performed gender conform or neutral sports (Table 3). Thereby, boys from single-sex PE settings mostly practiced male-classified sports. Half of them reported mostly playing soccer in PE lessons. Further sports included basketball, hockey, parkour, track and field and occasional volleyball as this student reported: “So we play soccer—a lot, but we also do other things like basketball and volleyball,.. yes, and that’s all we do. Otherwise sometimes like athletics, like running and throwing.” (47m_si). Some boys in single-sex settings reported that they have never practiced a non-gender conform sport. It seemed that they did not see the necessity since the classes were divided based on gender, as this boy explained: “We haven’t had that yet [non-gender conform sports], honestly, but we also have separate physical education classes, so boys and girls are separated.” (54m_si).

In general, girls from single-sex PE settings showed a higher variety of sports and practiced more gender-neutral and male-classified sports than did boys from single-sex PE settings. However, this seemed to depend on the teacher and school. In one school, they practiced mostly volleyball, gymnastics and fitness and one student described that they practiced “rather female sports” (6f_si). Another student from the same school wished for more diversity in their PE lessons:

“I think with those [gender] typical sports, it‘s sometimes just annoying because we never play soccer.. I think it would be good if we could do it more often.. then it might change and not always stays like this, that boys play soccer and girls dance.”

(4f_si)

In the other school, they showed a higher variety of performed sports and girls also practiced soccer, track and field, basketball, hockey, dance and lacrosse.

Whereas ballet and gymnastics were most often named as female sports, further descriptions of female sports differed. In coeducational settings, a sport was often attributed to a gender depending on the representation of male or female participants (Table 3), as can be seen in the following quotes by two male students from either a single-sex or coeducational PE setting: “Typical boys’ sports are soccer, typical girls’ sports, I don’t know, there are many in our class who play field hockey, I would say field hockey.” (20m_co); “So for me volleyball is a typical girl sport […] We are separated, we don‘t do sports with girls. Only this one time and there we played volleyball. Girls.. well with us [the boys] the girls belong to the low people, they are not so.., well for me,… girls, they just can‘t do anything, unlike the boys, the boys can do a lot more… “ […] (57m_si).

##### Teachers’ Views

In general, teachers said that girls were more open to male-classified sports and that it was easier for girls to participate in non-gender conform sports than it was for boys. Boys would be less open and less motivated to participate in female-classified sports. Whereas, coeducational teachers integrated female-classified sports in PE and had different approaches if resistance occurred as the following quote illustrates:

“It [performing non-gender conform sports] actually always works quite well. When you do it with dancing, for example, when you participate as a [male] sports teacher, then it’s good for the boys to see right away, ‘ok, he’s doing it too, then we’ll do it too, because it seems to be normal’…and what also works is when you have examples or show videos where boys or men are dancing and they see that it looks quite good and that [method] works quite well.”

(Teacher 4m_co)

Single-sex educational teachers, especially for boys, did not integrate non-gender conform sports, as can be seen in the following quote: “I’ve never got the boys to start dancing. […] They just slide into the [gender] role if you only have boys. You can try badminton, but it won’t work well,.. after 10 min they just say it’s too boring […] Then we play soccer [laughs].” (Teacher_9m_si).

#### 3.2.3. Reported Difficulties

More girls than boys reported difficulties with the opposite sex in PE lessons. Most reported difficulties were named as being excluded from ball games because boys would not want to pass the ball to the girls. However, of the eight students reporting difficulties, seven students were from single-sex classes. Their experiences were based on occasionally mixed classes (e.g., if a teacher was sick) and all referred to ball games or team sports. One student explained the occurring situation as following:

“Yes, [..] well, they see us as weaker and when we played something, for example, basketball, they often didn’t pay attention to the girls, only to the boys, because they thought, when they interact with the girls, that they lose or so.. you are downgraded, so that you are weaker, that annoyed me.”

(60f_si)

##### Teachers’ Views

Coeducational teachers did not report any general difficulties in teaching coeducational PE. Only one male coeducational teacher explained that he separated the PE class according to sex in swimming. This was done beforehand to avoid possible conflicts with religious reasons of some girls as is explained in the following quote:

“I only did it once [separating PE lessons by sex] in swimming lessons because we have two groups anyway and the situation was that the class was very heterogeneous and there were also many… [short pause] with Muslim faiths in the class and in order not to have any additional discussion or difficulties, we separated the group into boys and girls for swimming, which worked quite well.”

(Teacher 4m_co)

#### 3.2.4. Gender-Sensible Teaching

None of the teachers described talking about gender bias or gender stereotypes explicitly in PE classes; however, coeducational PE teachers were aware of different gender roles and addressed this issue if difficulties concerning non-gender conform sports arose, as this teacher described:

“I do talk about it [gender roles] sometimes, especially if there is resistance [towards an activity] or something like ‘uh, not soccer again or not dancing—we don’t have to do that’, of course I talk to them about it and we talk about it. But thematically, so that I say we’ll deal with it today, I’ve never done that. So in conversation, depending on the situation that I have to say something about it, then I do it.”

(Teacher 7f_co)

Teachers named time and knowledge as reasons for not addressing gender issues in PE. They described that they rather wanted to use the little time they had in PE for physical activity and not theoretical input which would result in more sedentary time for students. Knowledge regarding gender-sensible teaching was a further reason since none of the PE teachers described to have gender-specific knowledge in teaching PE classes. One teacher of the single-sex PE setting described that she was not aware of her influence on possibly deepening stereotypical behavior in students by performing certain sports (e.g., gender conform sports) or their role in constructing gendered behavior. She reported that “Honestly, I haven’t thought about it…” and said that she did not do gender conform sports on purpose, that it rather happened and was not reflected: “Probably it’s more because you want to be considerate because it’s a girl and you don’t play wild ball games, I can imagine that [in my case], but not consciously in that sense. I think it happens automatically. You always have girls and then you do girl-specific things.” (Teacher 2f_si).

This teacher explained that she thought that the PE lessons would look different in a coeducational form; that it “would probably be adapted more to bring the boys down a bit and push the girls a bit, but I think it would look different, the physical education” (Teacher 2f_si).

Although coeducational teachers did not report gender-specific knowledge, they reported being aware of different interests and attitudes and reported how they integrated non-gender conform sports into their classes and hoped that socialization processes would happen automatically. All coeducational teachers purposely tried to form mixed-gender groups to enhance participation, teamwork and group processes between both genders as the following quotes illustrate: “Simply that they learn that it is about together and not against each other.” (Teacher 1m_co).

“We also work a lot with group work in physical education or teams have to be formed. At the beginning, I always take over, but then I try to educate the classes so that they do it on their own, and that is of course a very important process. You also make sure that they are mixed at some point, depending on the sport, of course, but I always say in general: you have to make sure that there are both boys and girls in a group and then they start to communicate with each other. Then boys talk to girls who have totally avoided each other before and talk to each other and you don’t have that possibility in other subjects.”

(Teacher 6m_co)

Through possible habituation of coeducational PE classes, gender perceptions and gender roles of children might have changed throughout the last years as this coeducational teacher explained: “I think … that they take it much more … naturally that they have to do sports together. They are used doing sports together. When I think of the past, it was rather a bit embarrassing to do exercises together with girls or so and today it is more… almost like normal.” (Teacher 4m_co).

However, a male single-sex teacher could not see a change in gender roles in his school: “Actually, very stereotypically like a few years ago, the girls tend to rhythmic gymnastic movements, … so dancing and gymnastics, while boys rather, … Yes, I stay with the expression like the macho sports such as soccer, yes, these physical sports more and they are actually still… the [gender] role has not changed much there.” (Teacher 9m_si).

## 4. Discussion

This study aimed to assess the experiences of students and teachers from single-sex and coeducational PE settings to get a better understanding of the gendered experiences and implementation of gender aspects in different PE settings. Students from single-sex and coeducational PE settings reported differently regarding perceived gendered behavior and activities performed. Students from coeducational PE settings reported more gender-neutral behavior and a higher variety of performed activities in PE, including various non-gender conform activities. Neither students from coeducational nor most students from single-sex educational PE classes wanted to change their PE settings.

The obtained results could show the advantages of coeducational PE settings in terms of more gender-neutral behaviors and a wider variety of performed activities compared to single-sex PE settings. Due to the methodological approach of the study, no causal relationships could be drawn, but referring to the literature, the choice of activities might be important for a successful, coeducational PE and a higher MVPA [19,20]. Mixed-gender activities (e.g., Korfball) were shown to affect activity levels positively [18,20]. Further, introducing technical tasks for male- and female-gendered activities resulted in a higher situational interest of boys and girls in a coeducational physical education setting [19]. Previous research has shown that, especially for girls, coeducational PE settings had disadvantages resulting in lower well-being [5,17]. Most of those previous results were cross-sectional studies and did not take teaching behavior into account. However, it is widely discussed that coeducation often resulted in PE which is characterized by a traditional male context [33]. Further, gendered processes often happen unconsciously and could be reinforced by students’ and teachers’ behaviors [34,35]. Thus, teachers’ behavior and game forms might be crucial for an inclusive PE lesson [20,35]. Single-sex PE students of the present study reported mostly participating in gender conform activities with boys only participating in gender conform activities. Especially, closed male sports groups are discussed as a possible reinforcing factor for homophobia [8,36]. Coeducational classes are named by LGBTQ+ students as a possibility to weaken the binary gender division between girls and boys and a possibility to make PE classes more inclusive [8]. In another qualitative study, homosexual students reported how they disliked and rejected PE due to the construction and reinforcement of gender roles and gender stereotypes [37]. Those experiences were often made when the participants did not conform to typical masculine sports (e.g., not liking football but doing ballet); families further reported how their kids used to love PE till their homosexuality changed their experience of PE and made them feel excluded and insecure [37]. Queer identity was not mentioned by any of the interviewed students in the present study.

The interviewed students from coeducational classes in Germany described having participated in various non-gender conform activities such as soccer, football or dance and rhythmic activities. Contrary to a qualitative analysis from Germany where male German PE teachers did not report integrating rhythmic activities [38], male and female coeducational PE teachers confirmed students’ descriptions of rhythmic activities in PE lessons. Although coeducational teachers in the present study integrated non-gender conform activities, they only reported verbally addressing gender issues if resistance to the activities occurred. Teachers reported that they were missing the time and/or knowledge to thematically address gender issues. However, reflecting on gendered experiences in PE might lead to a more comprehensive experience and the dismantling of gender segregation by a joint reflection on PE experiences from male and female students’ points of view, as was shown in a previous study [6]. In a university sample of PE education teachers, McVeigh and Waring [39] observed and interviewed male and female students who took part in a 10-week gymnastic course. Gender roles and gendered practices were visible in didactic and autonomous teaching practices. Thus, the authors concluded that in order to challenge gender binaries a further discourse has to take place [39]. In the present study, students from coeducational classes reported participating in a wide variety of physical activities compared to students from single-sex PE. The type of activity could also influence PA levels, as was shown previously [18].

In the present study, students also mentioned some of the difficulties of coeducational PE. Difficulties were mostly reported by girls, such as complaints regarding being excluded in team sports (e.g., not getting the ball passed). Although this was reported more by girls from single-sex than coeducational PE settings, this is in line with previous analyses from coeducational PE settings and should be taken into account [35,38,40]. To address those difficulties in terms of limited participation in team sports, modified game forms could enhance participation and activity levels [20]. Forestier and Larsson [41] assessed if dance was a possibility to challenge gender norms in coeducational PE. Although girls dominated the dance lessons, stereotypical gender roles were reproduced and visible as there was a predefined male and female part in dancing [41]. In the present study, students and teachers talked about dance forms that were performed in a group and did not assign different dance parts based on gender but resulted in a group choreography (e.g., jumpstyle).

The opportunity for social learning, especially through coeducational PE settings, was mentioned by coeducational teachers only and was addressed by purposefully mixing teams. Social interaction was one of five themes identified as a central theme for a meaningful experience in PE [42]. Other themes were named as ‘fun’, ‘challenge’, ‘motor competence’ and ‘personally relevant learning’. In a recent study, it was shown that perceived motor competence had a higher influence on motivation for PE than actual motor competence [43].

Most of those themes identified in PE as meaningful experiences were also found in adventure sports participants as a motive for sports participation [44]. Adventure sports activities, such as skateboarding, climbing, mountain biking and freeriding, are highly popular among the youth [45] and provide the opportunity to fulfill the basic psychological needs of autonomy, competence and relatedness [46]. Research also showed the possibilities of those sports transgressing traditional gender roles [47,48,49]. Sport pedagogic researchers explained that physical education should draw from youth culture in order to be more relevant to students [50]. Further, adventure sports might address several developmental aspects in adolescents such as adolescents’ need for sensations and autonomy [46,51]. Regarding lifetime sports participation and the sports socialization process, sports experiences in late adolescence including school programs seem to play a crucial role [52]. Needs satisfaction and autonomous motivation, which are also characteristics named in adventure sports [46], were shown to predict behavioral engagement and intention in PA [53]. Since coeducational classes in the present study reported practicing a higher variety of different physical activities, the chance to find a likable activity that can be participated in the long-term and thus reach physical activity levels might be higher. However, future studies should investigate which activities could target socialization processes regarding gender attitudes, lifetime sports participation and thus contribute to reaching the physical activity recommendations.

### Strengths and Limitations

The most important strengths of this study are the large number of interviewed students with an equal gender distribution. However, readers should avoid generalizing the results of this study since a qualitative study is used to examine issues in great detail and depth [54] but cannot be used to make empirical statements over an entire population. Only students from three different higher education schools were interviewed. As also mentioned by one teacher in the results, the behavior of students might vary between schools. Even though the type of school was the same, we did not assess the socioeconomic status of participants although this is a further factor influencing sports participation [55].

## 5. Conclusions

Performed exercise activities and teacher’s behavior differed between coeducational and single-sex PE settings. Students from coeducational PE settings reported more neutral behavior and a higher variety of performed activities in PE including various non-gender conform activities. This might have influenced reported differences in terms of perceived gendered behavior and perceived gendered activities by students. None of the interviewed PE teachers reported having the time and/or knowledge to thematically and verbally address gender issues in PE settings. However, coeducational PE teachers purposefully formed mixed-sex groups and integrated non-gender conform activities to provide inclusivity and enhance group processes. They further reported verbally addressing gender roles if resistance to the activities occurred. Based on our results, training in gender awareness seems to be an important intervention for PE teachers. Further studies should evaluate the influence of specific physical activity interventions on gender perceptions and students’ behavior to give practical recommendations for PE classes.

## Figures and Tables

**Table 1:** Student characteristics divided into physical education settings.

	Single-Sex		Coeducation	
	Number	Age [Years] Mean (SD)	Number	Age [Years] Mean (SD)
Student female	17	14.2 (0.34)	17	13.4 (0.66)
Student male	16	14.6 (0.22)	14	13.1 (0.69)

**Table 2:** Descriptions of stereotypical behavior in PE attributed to either the female gender, male gender or no gender.

Subthemes	Code	Class Comparison	Gender Comparison
Female behavior	Reserved/cautious	coed = single-sex	females > males
	Afraid to get hit by a ball	coed > single-sex	females > males
	Not motivated	single-sex > coed	females > males
Male behavior	Poser	single-sex > coed	males > females
	Loud	single-sex > coed	males > females
	Aggressive	single-sex > coed	males > females
	Egoistic	coed = single-sex	females > males
No gendered behavior	Neutral gender behavior	coed > single-sex	females > males

**Table 3:** Overview of the subthemes perceived in gendered sport activities with class and gender comparison.

Subtheme	Code	Class Comparison	Gender Comparison
Practiced sport activities	Gender conform	single-sex > coed	males > females
	Non-gender conform	coed > single-sex	females > males
	Gender-neutral	coed > single-sex	males > females
Perceived male activities	Soccer	coed = single-sex	males > females
	Basketball	single-sex > coed	females
Perceived female activities	Volleyball	single-sex > coed	males > females
	Floorball	coed	males
	Rope skipping	coed	males
	Horseback riding	coed	females
	Hockey	coed	males
	Ballet	coed > single-sex	males > females
No gendered activities	Indifferent	coed > single-sex	males > females

## Data Availability

The data are not publicly available due to ethical considerations on preserving the anonymity of study participants.

## References

[1] Ekelund U., Steene-Johannessen J., Brown W.J., Fagerland M.W., Owen N., Powell K.E., Bauman A., Lee I.-M. (2016). Does physical activity attenuate, or even eliminate, the detrimental association of sitting time with mortality? A harmonised meta-analysis of data from more than 1 million men and women: A harmonised meta-analysis of data from more than 1 million men and women. Lancet.

[2] McPhie M.L., Rawana J.S. (2015). The effect of physical activity on depression in adolescence and emerging adulthood: A growth-curve analysis. J. Adolesc..

[3] Bull F.C., Al-Ansari S.S., Biddle S., Borodulin K., Buman M.P., Cardon G., Carty C., Chaput J.-P., Chastin S., Chou R. (2020). World Health Organization 2020 guidelines on physical activity and sedentary behaviour. Br. J. Sports Med..

[4] Slingerland M., Borghouts L. (2011). Direct and indirect influence of physical education-based interventions on physical activity: A review. J. Phys. Act. Health.

[5] Lyu M., Gill D.L. (2011). Perceived physical competence, enjoyment and effort in same-sex and coeducational physical education classes. Educ. Psychol..

[6] Sánchez-Hernández N., Martos-García D., Soler S., Flintoff A. (2018). Challenging gender relations in PE through cooperative learning and critical reflection. Sport Educ. Soc..

[7] Brown C.S., Stone E.A. (2016). Gender Stereotypes and Discrimination: How Sexism Impacts Development. Adv. Child Dev. Behav..

[8] Berg P., Kokkonen M. (2022). Heteronormativity meets queering in physical education: The views of PE teachers and LGBTIQ+ students. Phys. Educ. Sport Pedagog..

[9] Bussey K., Bandura A. (1999). Social cognitive theory of gender development and differentiation. Psychol. Rev..

[10] Dempster S. (2009). Having the balls, having it all? Sport and constructions of undergraduate laddishness. Gend. Educ..

[11] Adá-Lameiras A., Rodríguez-Castro Y. (2021). Analysis from a gender perspective of the Olympic Games on Twitter. Eur. Sport Manag. Q..

[12] Brown W.J., Mielke G.I., Kolbe-Alexander T.L. (2016). Gender equality in sport for improved public health. Lancet.

[13] Messner M.A. (2002). Taking the Field: Women, Men, and Sports.

[14] Chalabaev A., Sarrazin P., Fontayne P., Boiché J., Clément-Guillotin C. (2013). The influence of sex stereotypes and gender roles on participation and performance in sport and exercise: Review and future directions. Psychol. Sport Exerc..

[15] Schmalz D.L., Kerstetter D.L. (2006). Girlie Girls and Manly Men: Chidren′s Stigma Consciousness of Gender in Sports and Physical Activities. J. Leis. Res..

[16] Wallace L., Buchan D., Sculthorpe N. (2020). A comparison of activity levels of girls in single-gender and mixed-gender physical education. Eur. Phys. Educ. Rev..

[17] Mutz M., Burrmann U. (2014). Sind Mädchen im koedukativen Sportunterricht systematisch benachteiligt?. Sportwiss.

[18] McKenzie T.L., Prochaska J.J., Sallis J.F., LaMaster K.J. (2004). Coeducational and single-sex physical education in middle schools: Impact on physical activity. Res. Q. Exerc. Sport.

[19] Lentillon-Kaestner V., Roure C. (2019). Coeducational and single-sex physical education: Students’ situational interest in learning tasks centred on technical skills. Phys. Educ. Sport Pedagog..

[20] van Acker R., Da Carreiro Costa F., de Bourdeaudhuij I., Cardon G., Haerens L. (2010). Sex equity and physical activity levels in coeducational physical education: Exploring the potential of modified game forms. Phys. Educ. Sport Pedagog..

[21] van Doodewaard C., Knoppers A. (2018). Perceived differences and preferred norms: Dutch physical educators constructing gendered ethnicity. Gend. Educ..

[22] Preece S., Bullingham R. (2022). Gender stereotypes: The impact upon perceived roles and practice of in-service teachers in physical education. Sport Educ. Soc..

[23] Joy P., Zahavich J.B.L., Kirk S.F.L. (2021). Gendered bodies and physical education (PE) participation: Exploring the experiences of adolescent students and PE teachers in Nova Scotia. J. Gend. Stud..

[24] Rief M., Oesterhelt V., Amesberger G. (2022). Education and professionalization of Physical Education Teachers: Research trends and developments in German-language literature in relation to Anglophone perspectives. Phys. Educ. Sport Pedagog..

[25] Feilzer M.Y. (2010). Doing Mixed Methods Research Pragmatically: Implications for the Rediscovery of Pragmatism as a Research Paradigm. J. Mix. Methods Res..

[26] Giacobbi P.R., Poczwardowski A., Hager P. (2005). A Pragmatic Research Philosophy for Sport and Exercise Psychology. Sport Psychol..

[27] Brown D.J., Arnold R., Reid T., Roberts G. (2018). A Qualitative Exploration of Thriving in Elite Sport. J. Appl. Sport Psychol..

[28] Marshall B., Cardon P., Poddar A., Fontenot R. (2013). Does Sample Size Matter in Qualitative Research?: A Review of Qualitative Interviews in is Research. J. Comput. Inf. Syst..

[29] Patton M.Q. (1990). Qualitative Research & Evaluation Methods.

[30] Hennink M., Hutter I., Bailey A. (2011). Qualitative Research Methods.

[31] Braun V., Clarke V., Weate P. (2016). Using Thematic Analysis in Sport and Exercise Research.

[32] Smith B., Sparkes A.C. (2009). Narrative analysis and sport and exercise psychology: Understanding lives in diverse ways: Understanding lives in diverse ways. Psychol. Sport Exerc..

[33] Mccaughtry N. (2006). Working politically amongst professional knowledge landscapes to implement gender-sensitive physical education reform. Phys. Educ. Sport Pedagog..

[34] Berg P., Lahelma E. (2010). Gendering processes in the field of physical education. Gend. Educ..

[35] Brown S., Macdonald D. (2008). Masculinities in Physical Recreation: The (re)production of masculinist discourses in vocational education. Sport Educ. Soc..

[36] Hartmann-Tews I., Braumüller B., Menzel T., Staritz N. Sexuelle Orientierung, Geschlechtsidentität und Sport: Ausgewählte Ergebnisse und Handlungsempfehlungen. www.out-sport.eu.

[37] Hortigüela-Alcala D., Chiva-Bartoll O., Hernando-Garijo A., Sánchez-Miguel P.A. (2022). Everything is more difficult when you are different: Analysis of the experiences of homosexual students in Physical Education. Sport Educ. Soc..

[38] Kastrup V., Kleindienst-Cachay C. (2016). ‘Reflective co-education’ or male-oriented physical education? Teachers’ views about activities in co-educational PE classes at German secondary schools. Sport Educ. Soc..

[39] McVeigh J., Waring M. (2021). Developing the pedagogical practice of physical education pre-service teachers in gymnastics: Exploring gendered embodiment. Phys. Educ. Sport Pedagog..

[40] Hills L.A., Croston A. (2012). ‘It should be better all together’: Exploring strategies for ‘undoing’ gender in coeducational physical education. Sport Educ. Soc..

[41] Forestier A., Larsson H. (2021). Choreographing gender: Masculine domination and heteronormativity in physical education. Sport Educ. Soc..

[42] Beni S., Fletcher T., Ní Chróinín D. (2017). Meaningful Experiences in Physical Education and Youth Sport: A Review of the Literature. Quest.

[43] Estevan I., Bardid F., Utesch T., Menescardi C., Barnett L.M., Castillo I. (2021). Examining early adolescents’ motivation for physical education: Associations with actual and perceived motor competence. Phys. Educ. Sport Pedagog..

[44] Frühauf A., Zenzmaier J., Kopp M. (2020). Does Age Matter? A Qualitative Comparison of Motives and Aspects of Risk in Adolescent and Adult Freeriders. J. Sports Sci. Med..

[45] Bennett G., Henson R.K., Zhang J. (2003). Generation Y’s Perceptions of the Action Sports Industry Segment. J. Sport Manag..

[46] Clough P., Houge Mackenzie S., Mallabon L., Brymer E. (2016). Adventurous Physical Activity Environments: A Mainstream Intervention for Mental Health. Sports Med..

[47] Dilley R.E., Scraton S.J. (2010). Women, climbing and serious leisure. Leis. Stud..

[48] Kelly D.M., Pomerantz S., Currie D. (2006). Skater girlhood and emphasized femininity: ‘you can’t land an ollie properly in heels’. Gend. Educ..

[49] Frühauf A., Pahlke C., Kopp M. (2022). Overcoming Gender Barriers in Sports—An Opportunity of Adventure/High Risk Sports?. Sociol. Sport J..

[50] Gard M., Hickey-Moodey A., Enright E. (2013). Youth culture, physical education and the question of relevance: After 20 years, a reply to Tinning and Fitzclarence. Sport Educ. Soc..

[51] Dahl R.E., Allen N.B., Wilbrecht L., Suleiman A.B. (2018). Importance of investing in adolescence from a developmental science perspective. Nature.

[52] Scheerder J., Thomis M., Vanreusel B., Lefevre J., Renson R., Vanden Eynde B., Beunen G.P. (2006). Sports Participation Among Females From Adolescence To Adulthood. Int. Rev. Sociol. Sport.

[53] Leo F.M., Mouratidis A., Pulido J.J., López-Gajardo M.A., Sánchez-Oliva D. (2022). Perceived teachers’ behavior and students’ engagement in physical education: The mediating role of basic psychological needs and self-determined motivation. Phys. Educ. Sport Pedagog..

[54] Anderson C. (2010). Presenting and evaluating qualitative research. Am. J. Pharm. Educ..

[55] Heradstveit O., Haugland S., Hysing M., Stormark K.M., Sivertsen B., Bøe T. (2020). Physical inactivity, non-participation in sports and socioeconomic status: A large population-based study among Norwegian adolescents. BMC Public Health.

